# Dark Tetrad traits and problematic generative artificial intelligence use: the serial mediating role of individualism and intolerance of uncertainty

**DOI:** 10.1186/s40359-026-05012-4

**Published:** 2026-06-13

**Authors:** Kağan Kırcaburun

**Affiliations:** 1 Educational Sciences Department, Düzce University, Düzce, Turkey; 2 Faculty of Education, Duzce University, Konuralp Campus, Duzce, 81620 Turkey

**Keywords:** Artificial intelligence, Individualism, Intolerance of uncertainty, Dark personality, Generative AI

## Abstract

**Background:**

With the rapid integration of generative artificial intelligence into daily life, understanding the personality-based factors underlying problematic usage patterns has become increasingly important.

**Objective:**

The present study investigated the relationships between dark personality traits (narcissism, Machiavellianism, psychopathy, and sadism) and problematic generative artificial intelligence use (PGAIU), with a particular focus on the serial mediating role of individualism and intolerance of uncertainty.

**Methods:**

Data were collected from a large adult sample of generative artificial intelligence (GAI) users (*N* = 677; 50% men; *M*_age_ = 39.87) using self-report instruments.

**Results:**

Path analysis revealed that narcissism was positively associated with PGAIU through both direct and indirect pathways, whereas sadism demonstrated a small but significant total effect without identifiable direct or indirect mechanisms. Vertical individualism and intolerance of uncertainty were consistently and positively related to PGAIU across the total sample and sex-based groups. Further analyses suggested that, among women, the association between narcissism and PGAIU was fully indirect, operating through vertical individualism. In addition, psychopathy showed a divergent pattern among men, with a direct negative association with PGAIU alongside an indirect positive association via intolerance of uncertainty-related pathways. These patterns should be interpreted with caution, as full measurement invariance across sex was not supported.

**Conclusion:**

Overall, the findings highlight the central role of individualistic value orientations and uncertainty-related processes in explaining how dark personality traits contribute to PGAIU. These results offer initial insights, within this sample and with these measures, into potential psychological mechanisms underlying dysregulated engagement with emerging GAI technologies. Replication in independent samples is needed before stronger conclusions can be drawn.

**Supplementary Information:**

The online version contains supplementary material available at 10.1186/s40359-026-05012-4.

## Introduction

Generative artificial intelligence (GAI) has rapidly become central to both individual and institutional domains. Its transformative impact is now frequently compared to earlier technological breakthroughs like the internet and mobile devices [1]. Unlike earlier AI systems that focused on classification or prediction, current GAI tools (e.g., ChatGPT) can generate creative and personalized content almost instantaneously [2]. While these features have led to wide adoption for work and personal purposes [3], they also create new risks. Specifically, the high accessibility, immediacy, and personalization of GAI may heighten the risk of excessive and potentially problematic use [4]. Individual differences in personality may be particularly relevant in determining who is susceptible to such excessive use, as personality traits shape how individuals engage with and respond to novel technologies [5]. Accordingly, the present study focuses on personality-based pathways underlying problematic GAI use (PGAIU). In this context, it is important to first define what constitutes PGAIU.

PGAIU is typically characterized by difficulties controlling GAI-related behaviors to the extent that it begins to interfere with daily activities—such as academic performance, work tasks, and interpersonal relationships—and is accompanied by withdrawal-like distress, preoccupation, and continued use despite negative consequences [5, 6]. While acknowledging that PGAIU is a construct still under conceptual development and that its theoretical status as a behavioral addiction remains to be confirmed empirically, the present study adopts an addiction-like framework because the patterns described above are consistent with core criteria widely used in behavioral addiction research (e.g., salience, mood modification, withdrawal, conflict, and relapse; [7]). This framing is not intended to imply clinical disorder, but rather to provide a theoretically grounded basis for studying disproportionate and dysregulated GAI engagement.

Current empirical work on PGAIU has increasingly shifted toward understanding the psychological mechanisms underlying this behavior. So far, PGAIU has been associated with a range of adverse correlates, such as academic difficulties, cognitive impairments, psychosocial maladjustment, maladaptive technology-related behaviors, and poorer mental health [4–6, 8–10]. Despite these important findings, two notable gaps warrant attention. First, a substantial portion of existing PGAIU research has been conducted in East and Southeast Asian contexts (e.g., China, Singapore, Taiwan; [4–6, 9]), with comparatively fewer studies drawing on Western community samples. This geographic skew is noteworthy because individualism-collectivism differences between Eastern and Western societies may shape the psychological mechanisms linking personality to technology use [11]. Examining these associations in a Western adult sample therefore constitutes a theoretically meaningful contribution.

Second, although dark personality traits, individualistic value orientations, and intolerance of uncertainty have each been independently implicated in problematic technology use, no prior study has examined these constructs together within a single mediation model. This gap is particularly significant because the I-PACE framework [12] predicts that person-level variables (such as dark traits) shape problematic technology use through their influence on affective and cognitive processes. The present study directly addresses this by proposing dark traits as distal predictors, individualism as a value-based mediator reflecting motivational orientation, and IU as a proximal cognitive-affective mediator. This sequential logic is theoretically grounded and distinguishes the present model from simpler bivariate approaches.

The Interaction of Person–Affect–Cognition–Execution (I-PACE) model provides a broad organizing framework for these variables. In the present study, I-PACE is used primarily as a heuristic framework to contextualize the proposed associations rather than to increase model complexity. The I-PACE model posits that problematic technology use develops through the interplay between person-level characteristics (P; e.g., personality traits, biological predispositions) and affective-cognitive responses (A-C; e.g., emotional reactions, cognitive appraisals), which together influence executive (E) decisions to engage with technology [12]. Personality traits are particularly relevant at the P level: they constitute stable individual difference variables that can shape both motivational orientations and cognitive-affective responses to technology affordances, thereby influencing susceptibility to dysregulated use [5].

Within this framework, antisocial personality traits such as the Dark Tetrad may foster stronger individualistic orientations [13], reflecting the self-serving and agentic motivational style that characterizes these traits. These individualistic orientations, in turn, may give rise to elevated intolerance of uncertainty (IU), as individuals who are primarily self-reliant have fewer social resources to buffer ambiguity [14]. IU can be conceptualized as an affective–cognitive response to ambiguous situations [15], which may increase reliance on GAI as a readily available tool for reducing uncertainty and negative affect. Accordingly, the present study examines the serial mediating roles of individualism and IU in the relationship between Dark Tetrad traits (narcissism, Machiavellianism, psychopathy, and sadism) and PGAIU. This perspective is also consistent with innovation adoption frameworks, which emphasize the role of individual characteristics, such as personality, in the adoption and continued use of emerging technologies [16].

### Dark Tetrad and PGAIU

Empirical research examining personality-related risk factors for PGAIU remains notably scarce. Initial findings suggest that openness, agreeableness, conscientiousness, and extraversion are negatively correlated with PGAIU [5], yet the role of maladaptive and socially aversive traits remains largely unexplored. Only one very recent study has demonstrated that antagonistic traits (i.e., narcissism, Machiavellianism, psychopathy) are linked to PGAIU through moral disengagement and unethical GAI use [17]. However, the observed effects were only partially mediated, suggesting that additional psychological mechanisms may contribute to the association between dark personality traits and PGAIU. The Dark Tetrad traits may offer a more detailed explanation for PGAIU vulnerability through their links to deficient self-regulation and atypical reward sensitivity [18]. These characteristics align with the Person-level (P) variables of the I-PACE framework [12] and have been consistently linked to problematic use of internet, social media, and online gaming [19–21]. Unlike conventional digital tools, GAI provides instantaneous, highly personalized, and low-effort outputs [4], features that may be especially appealing to individuals with dark traits and encourage uncontrolled use.

Machiavellianism’s strategic orientation may prompt users to view GAI as an instrument for impression management or deceptive advantage [22]. Likewise, narcissism—driven by grandiosity and validation needs—may engage with GAI to project inflated status or competence with minimal effort [23]. For psychopathy, immediate feedback and low-effort gains may further compromise already weakened behavioral inhibition and amplify sensation seeking [24]. Sadism introduces a distinct dimension: GAI’s capacity to generate harmful or exploitative content may provide an accessible outlet for deriving pleasure from dominance or others’ discomfort [25]. Such patterned engagement may strengthen reinforcement processes over time, heightening the risk of problematic use [12]. Accordingly, we anticipate that all Dark Tetrad traits will show positive associations with PGAIU.

### The mediating role of individualism

The distinction between individualism and collectivism was originally proposed to understand how societies prioritize personal autonomy versus group cohesion [11]. While collectivism involves defining the self through group relations, individualism emphasizes independence and self-reliance [26]. These constructs were later refined into vertical and horizontal forms [14]. More specifically, vertical individualism emphasizes competition and status hierarchy, whereas horizontal individualism reflects a preference for uniqueness alongside an emphasis on equality [26]. In the present study, we focused exclusively on individualism, as the agentic and self-serving nature of the Dark Tetrad aligns more closely with these values than with collective orientations [27]. Importantly, in the present study, individualism is conceptualized as an individual-level value orientation rather than a country-level cultural dimension.

From a dispositional perspective, each Dark Tetrad trait includes characteristics that are conceptually and motivationally aligned with individualistic value orientations. Narcissism is characterized by grandiosity, a strong sense of entitlement, and a persistent need for admiration, along with an agentic orientation toward self-promotion and status attainment—features that closely correspond to vertical individualism [27]. Machiavellianism reflects strategic self-interest and a willingness to exploit social relationships for personal gain, suggesting an individualistic orientation in which others are treated instrumentally rather than as relational partners [13, 28]. Psychopathy, defined by callousness, shallow affect, and reduced prosocial motivation, is associated with lower communal orientation and disengagement from the reciprocal obligations that characterize collectivist norms [26, 28]. Although empirical research directly linking sadism to individualistic values is limited, sadistic individuals’ tendency to seek dominance and derive pleasure from others’ suffering reflects a self-focused orientation that is inconsistent with group-oriented values [29]. While previous research has mainly linked individualism to traits such as neuroticism and openness [30], the self-enhancing, exploitative, and socially disengaged features of the Dark Tetrad suggest that these traits may collectively promote a worldview centered on personal advantage rather than collective belonging. Accordingly, each Dark Tetrad trait is expected to be positively associated with individualistic orientations.

There is growing empirical support for individualism as a significant contributor to maladaptive technology use. For example, general individualism has been positively related to internet addiction via the frustration of basic psychological needs [31], while vertical individualism has been linked to cyberbullying via the mediating role of internet addiction [32]. Individualistic tendencies have also been associated with higher levels of social media and video game addictions [33], suggesting that this value orientation constitutes a transdiagnostic vulnerability factor across multiple forms of problematic digital behavior. GAI tools, by virtue of their capacity to deliver rapid, high-quality, and highly personalized outputs, are particularly well-suited to serving the efficiency-maximizing and goal-achieving motivations of individualistic users [14]. Vertical individualists, who are especially motivated to outperform peers and signal competence, may be drawn to GAI as a performance-amplifying resource in competitive professional or academic contexts [24]. Over time, the repeated instrumental use of GAI to fulfill status and achievement goals may habituate into patterns of excessive and uncontrolled use. Accordingly, we expect that individualism—and vertical individualism in particular—will function as a value-based motivational mediator linking Dark Tetrad traits to PGAIU.

### The mediating role of intolerance of uncertainty

Intolerance of uncertainty (IU) refers to a dispositional tendency to experience discomfort and distress when facing uncertain situations or ambiguous outcomes, even when the probability of negative consequences is low [15]. This construct is recognized as a central cognitive vulnerability underlying excessive worry and heightened anxiety responses [34]. Recent evidence indicates that dark personality traits collectively explain a significant proportion of variance in IU [35], with narcissism, Machiavellianism, and psychopathy each showing unique positive associations with higher levels of uncertainty intolerance [36, 37]. These findings suggest that individuals high in dark traits may experience increased emotional strain and maladaptive stress responses, reducing their capacity to cope effectively with uncertainty [38].

Although direct empirical evidence remains limited, sadism may also be theoretically linked to IU, as individuals high in sadistic traits tend to seek dominance and control [29]—characteristics that are challenged under conditions of ambiguity. Individualism may also be positively related to IU. Highly individualistic individuals rely more on the self than on social support when facing uncertainty [14]. Because they are more likely to avoid close interpersonal relationships and withdraw from social environments [39], they may have fewer external resources to buffer uncertainty-related stress. Furthermore, a tendency to avoid shared responsibility or collaborative problem-solving may limit adaptive coping strategies [40], thereby increasing distress when outcomes are unclear.

In recent years, IU has been increasingly included in models of problematic technology use. For example, IU predicts smartphone addiction even after controlling for depression, anxiety, and cyberchondria [41]. Likewise, the association between IU and problematic social media use is mediated by maladaptive coping and fear of missing out [42]. These findings suggest that problematic technology use may function as a maladaptive strategy to manage the stress and rumination arising from elevated IU [43]. From this perspective, GAI may represent a particularly attractive solution for individuals with high IU, as it can be used to cope with negative emotional states [5]. Elevated IU often impairs problem-solving ability, leading to inaction and avoidance when individuals encounter ambiguous situations [44]. GAI can help users complete tasks quickly, support decision-making, and reduce uncertainty in performance situations [2]. Over time, this reliance may increase and place individuals at greater risk of developing dependent or problematic use patterns.

Guided by this rationale, we propose a serial mediation model grounded in the I-PACE framework. Specifically, we expect that Dark Tetrad traits will foster stronger individualistic orientations, which in turn will increase intolerance of uncertainty by limiting social buffering and adaptive coping, ultimately elevating the risk of PGAIU. Therefore, we expect that individualism and intolerance of uncertainty will serially mediate the relationship between Dark Tetrad traits and PGAIU.

### The role of sex

Sex differences have long been considered an important factor in research examining dark personality traits and their associated psychological processes. Men generally display higher levels of Dark Tetrad traits than women [45, 46]. In addition, a recent meta-analysis showed that men are more strongly characterized by elevated individualistic orientations [47]. By contrast, women tend to report greater intolerance of uncertainty and higher anxiety levels [48], indicating distinct emotional and cognitive vulnerability patterns across sexes. Consistent with these differences, men also report higher levels of PGAIU and are more likely to engage in maladaptive patterns of GAI use than women [4, 49]. Taken together, these findings suggest that the pathways proposed in this study may operate differently for men and women.

Based on the aforementioned rationale, we hypothesized that Dark Tetrad traits would be both directly and indirectly associated with PGAIU via individualism and intolerance of uncertainty, with a serial pathway from individualism to intolerance of uncertainty. Beyond testing these direct and indirect effects, we also examined whether these associations differ between men and women. Finally, because younger age is a consistent risk factor for technology-related problematic behaviors [19], age was included as a control variable in the model.

## Methods

### Participants and procedure

Data collection was carried out via Prolific, an established online research recruitment platform that provides access to diverse and reliable participant pools. Prolific is widely used in behavioral and social science research due to its transparent participant prescreening, fair compensation practices, and high data quality standards. A growing body of independent research has consistently shown that samples obtained via Prolific exhibit strong data quality, broader demographic diversity, and higher replicability when compared to traditional student samples and other crowdsourcing platforms such as Amazon Mechanical Turk [50, 51]. Participants were recruited via Prolific using standard recruitment procedures without explicit quotas; however, the observed distribution of sex reflects the composition of the available participant pool and platform-level allocation mechanisms. Eligibility criteria included being an adult, being a native English speaker, and reporting regular use of generative AI (i.e., using GAI several times per week), as assessed via a self-report screening item at the beginning of the survey. The median completion time was 9 min and 23 s.

The final sample comprised 677 individuals who reported active use of generative artificial intelligence (GAI) tools (50% men; *M*_age_ = 39.87, *SD*_age_ = 13.41), with ages ranging from 18 to 83 years. The number of participants within each sex category exceeded the minimum sample size recommended for producing stable correlation coefficients (*n* ≥ 250), as suggested by Schönbrodt and Perugini [52]. Before taking part in the study, all participants were provided with detailed information regarding the study objectives and procedures. They were explicitly informed that participation was entirely voluntary and that their responses would be handled anonymously and kept strictly confidential. Informed consent was obtained electronically, with participants required to confirm that they had read and understood the study information prior to completing the survey. Ethical approval was granted by the relevant institutional ethics committee, and all study procedures were implemented in accordance with the ethical standards set out in the Declaration of Helsinki.

### Measures

At the outset of the survey, respondents reported basic demographic characteristics, namely age, sex, country of residence, level of education, relationship status, and the generative artificial intelligence (GAI) tool they used most frequently (see Table 1). Following the demographic section, participants completed a series of standardized self-report instruments assessing problematic GAI use (PGAIU), dark personality characteristics, individualism, and intolerance of uncertainty.

**Table 1:** Participants’ demographic characteristics

Variable		*N*	%
Sex			
	Men	339	50.0
	Women	337	50.0
	Other	1	0.0
Country			
	United States of America	274	40.5
	United Kingdom	175	25.8
	Canada	133	19.6
	Australia	55	8.1
	Other	40	5.9
Education			
	Undergraduate	335	49.5
	High school or equivalent	206	30.4
	Master’s degree	117	17.3
	Phd	17	2.5
	Primary school	2	0.3
Relationship status			
	In a relationship	419	61.9
	Not in a relationship	258	38.1
AI tools used			
	ChatGpt	454	67.1
	Google Gemini	105	15.5
	Microsoft Copilot	55	8.1
	X Grok	19	2.8
	Other	18	2.7
	Claude	16	2.4
	Perplexity	10	1.5

### Problematic GAI Use Scale (PGAIUS)

PGAIU was measured using the PGAIUS [6]. Although the original instrument was designed to assess problematic use of conversational artificial intelligence, all item references were adapted to explicitly refer to GAI in the present study, ensuring conceptual alignment with the research focus. The scale comprises six statements rated on a five-point (1 = *very rarely*; 5 = *very often*) Likert response format (e.g., “*I tried to cut down on the use of GAI without success*”). Mean scores across the items were computed to yield a single overall indicator of PGAIU, with higher values reflecting greater levels of problematic GAI use. A confirmatory factor analysis has been conducted to examine the factor structure of the adapted scale, and the results support its structural validity (χ² = 31.59, df = 7, *p* < .001, RMSEA = 0.07, 90% CI [0.05, 0.10], SRMR = 0.03, CFI = 0.99, GFI = 0.98). Information regarding the instrument’s psychometric properties— namely internal consistency, reliability, and evidence of convergent and discriminant validity (Cronbach’s alpha, McDonald’s omega, average variance extracted [AVE], and composite reliability [CR])—is presented in Table 2.

**Table 2:** *Mean scores*,* standard deviations*,and Pearson’s correlations of the study variables (*N* = 677)

	ω	CR	AVE	α	1	2	3	4	5	6	7	8	9	10
1. Problematic Gen-AI use	0.85	0.89	0.58	0.85	-									
2. IU	0.92	0.93	0.53	0.92	0.35***	**-**								
3. IU_Prospective	0.88	0.91	0.59	0.88	0.32***	0.95***	-							
4. IU_Inhibitory	0.83	0.88	0.59	0.82	0.35***	0.95***	0.80***	-						
5. Horizontal individualism	0.84	0.89	0.66	0.83	0.01	0.08*	0.04	0.11**	-					
6. Vertical individualism	0.76	0.84	0.57	0.74	0.41***	0.20***	0.15***	0.24***	0.21***	**-**				
7. Narcissism	0.86	0.89	0.67	0.86	0.29***	0.16***	0.17***	0.14***	− 0.05	0.45***	**-**			
8. Machiavellianism	0.85	0.81	0.52	0.85	0.19***	0.17***	0.20***	0.13**	− 0.03	0.25***	0.46***	**-**		
9. Psychopathy	0.81	0.86	0.61	0.81	0.07	0.20***	0.21***	0.17***	0.04	0.18***	0.29***	0.60***	**-**	
10. Sadism	0.69	0.91	0.52	0.70	0.12**	0.12**	0.13**	0.10*	0.07	0.16***	0.14***	0.31***	0.36***	-
11. Sex					0.05	− 0.06	− 0.08	− 0.05	− 0.05	0.12**	0.04	0.07	0.16***	0.06
12. Age					− 0.21***	− 0.19***	− 0.18***	− 0.18***	0.06	− 0.14***	− 0.12**	− 0.12**	− 0.05	− 0.08*
*Mean score*					1.86	5.61	2.80	2.81	7.31	4.76	3.64	3.36	2.70	1.06
*Standard deviation*					0.76	1.75	0.91	0.93	1.27	1.64	1.84	1.79	1.58	0.12
*Skewness*					0.99	0.15	0.18	0.09	− 0.85	0.09	0.30	0.39	0.97	3.67
*Kurtosis*					0.78	− 0.62	− 0.69	− 0.72	1.11	− 0.39	− 0.73	− 0.82	0.45	18.02

Note. Ꞷ = McDonald’s Omega reliability coefficient, *CR *Composite reliability, *AVE *Average variance extracted, α  Cronbach’s alpha reliability coefficient, G*en-AI *Generative artificial intelligence, *IU *Intolerance of uncertainty, Sex (coded as 1 = female, 2 = male)

* *p* < .05, ** *p* < .01, *** *p* < .001

### Intolerance of Uncertainty Scale (IUS-12)

Intolerance of uncertainty was assessed using the IUS-12 [15], which captures individual differences in responses to uncertain situations. The scale consists of two theoretically grounded dimensions: prospective anxiety (e.g., “*It frustrates me not having all the information I need.*”), reflecting cognitive concern and apprehension about future uncertainty, and inhibitory anxiety (e.g., “*The smallest doubt can stop me from acting.*”), representing behavioral inhibition and avoidance triggered by uncertainty. The IUS-12 comprises twelve items rated on a five-point Likert scale ranging from 1 (*not at all characteristic of me*) to 5 (*entirely characteristic of me*). Item scores were averaged, with higher values indicating greater intolerance of uncertainty. In the present study, both subscales demonstrated satisfactory reliability and construct validity. Detailed psychometric results are reported in Table 2.

### Individualism and Collectivism Scale (ICS)

Individualistic orientations were assessed using the ICS [14], a 16-item instrument developed to capture four distinct cultural dimensions: vertical collectivism, horizontal collectivism, vertical individualism (e.g., “*I’d rather depend on myself than others.*”), and horizontal individualism (e.g., “*It is important that I do my job better than others.*”), with each dimension represented by four items. In the present study, only the items corresponding to the individualism-related dimensions (vertical and horizontal individualism) were administered in order to specifically assess individualistic tendencies. Responses were recorded on a 9-point Likert scale ranging from 1 (*strongly disagree*) to 9 (*strongly agree*), with higher scores indicating stronger endorsement of individualistic values. Accordingly, individualism was conceptualized as an individual-level value orientation and measured using a self-report scale. The individualism subscales demonstrated acceptable reliability and construct validity in the current sample. Psychometric properties of the scale are reported in Table 2.

### Dark Triad Dirty Dozen Scale (DTDD)

Dark personality characteristics were measured using the DTDD scale developed by Jonason and Webster [53]. This instrument captures three fundamental personality dimensions: narcissism (e.g., “*I tend to expect preferential treatment from others*”), Machiavellianism (e.g., “*I have employed flattery to influence others*”), and psychopathy (e.g., “*I can be emotionally cold or insensitive*”). Each trait is operationalized through four items, with responses recorded on a nine-point Likert-type scale ranging from 1 (*strongly disagree*) to 9 (*strongly agree*). Analyses conducted in the present study indicated that all three subscales exhibited acceptable levels of reliability and construct validity within the current sample. Detailed psychometric findings are reported in Table 2.

### Short Sadistic Impulses Scale (SSIS)

Sadistic tendencies were measured using the SSIS developed by O’Meara et al. [29]. This instrument assesses everyday sadistic impulses, conceptualized as the propensity to derive pleasure from causing or observing harm or discomfort to others (e.g., “*I enjoy seeing people get hurt*”). The scale comprises ten items answered using a binary response format, with participants indicating whether each statement was unlike me (1) or like me (2). Item scores were summed to produce an overall index of sadistic tendencies, with higher total scores reflecting stronger sadistic impulses. In the current sample, the SSIS demonstrated acceptable levels of internal consistency and construct validity. Psychometric properties of the scale are presented in Table 2.

### Data analytic strategy

Prior to the main analyses, preliminary data screening procedures were conducted. Skewness and kurtosis values were examined to assess univariate normality. In addition, descriptive statistics and bivariate correlations were computed for all study variables to evaluate their distributions and initial associations, indicating that the data were suitable for subsequent parametric analyses. In addition, sex-based group comparisons were conducted using independent-samples *t*-tests. Measurement invariance was tested via using configural and metric invariance analyses. Potential common method bias was examined using Harman’s single-factor test, assessing whether a single latent factor accounted for the majority of the variance in the observed measures.

Model fit was assessed using multiple goodness-of-fit indices, namely the comparative fit index (CFI), goodness-of-fit index (GFI), root mean square error of approximation (RMSEA), and standardized root mean square residual (SRMR). Evaluation criteria for acceptable and good model fit were based on commonly cited methodological guidelines. Consistent with widely accepted guidelines, CFI and GFI values of 0.90 or above were considered indicative of acceptable model fit, with values of 0.95 or higher reflecting good fit. For SRMR, values below 0.08 were interpreted as acceptable. Regarding RMSEA, values ≤ 0.08 were taken to indicate acceptable fit, while values between 0.08 and 0.10 were interpreted as reflecting *marginal but tolerable fit*, particularly in complex models and large samples, as suggested in previous methodological work [54–56].

**Fig. 1:**
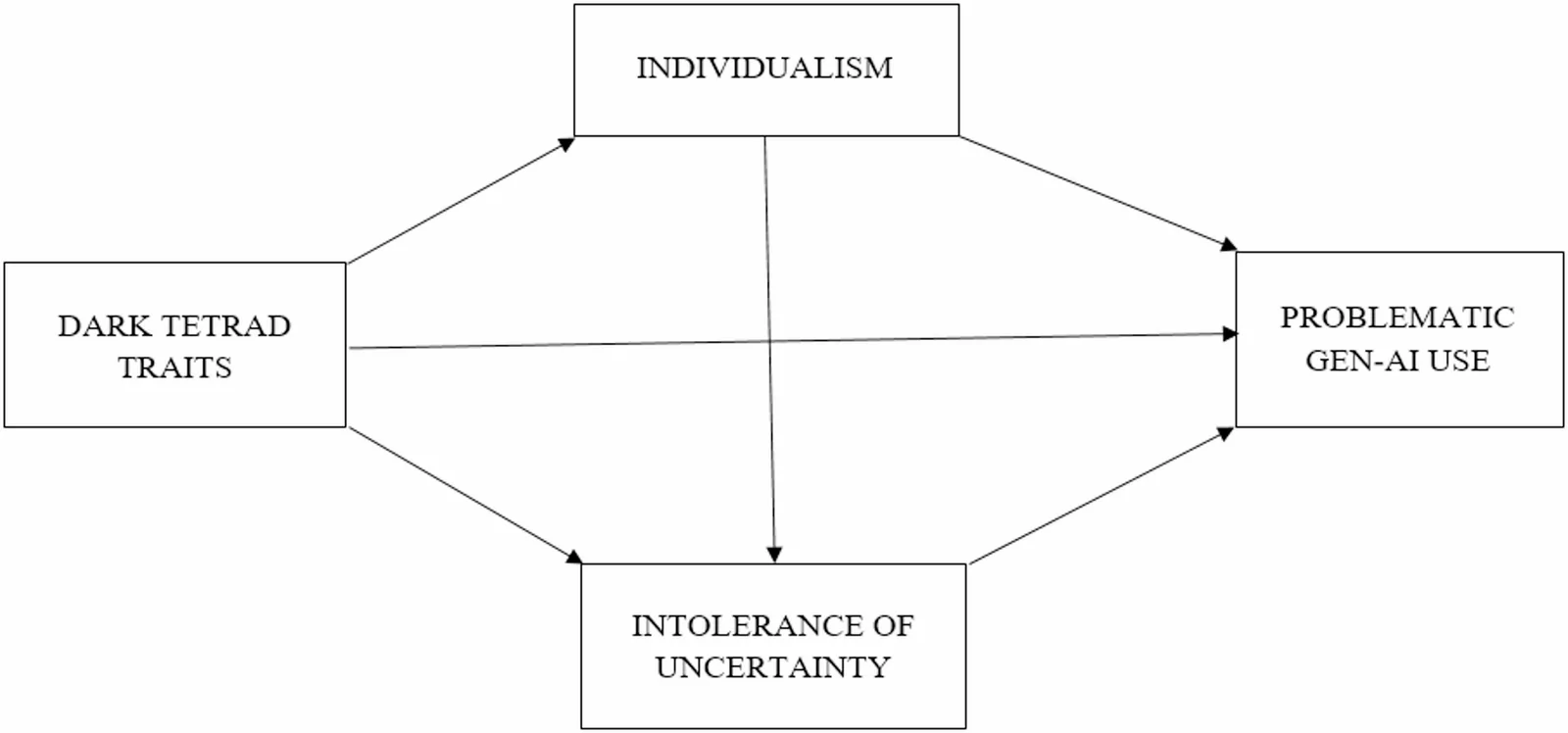
Theoretical model

Path analysis was conducted to examine the proposed direct and indirect associations among dark personality traits, individualism, intolerance of uncertainty, and PGAIU (see Fig. 1). The model was estimated using maximum likelihood (ML) estimation in AMOS. Given deviations from normality, bias-corrected bootstrap procedures with 10,000 resamples were employed to obtain robust confidence intervals for both direct and indirect effects, and statistical significance was evaluated based on 95% confidence intervals. Participant age and sex were entered as covariates in the model to control for their potential effects. Additionally, multi-group path analyses were performed to assess whether the relationships differed by sex. All statistical analyses were carried out using SPSS version 26 and AMOS version 24.

## Results

### Descriptive statistics

Table 2 presents the descriptive statistics, reliability estimates, and Pearson’s correlation coefficients for the study variables. Most scales demonstrated acceptable to excellent internal consistency, with Cronbach’s alpha and McDonald’s omega coefficients equal to or exceeding 0.74. The sadism scale exhibited marginal but acceptable internal consistency (α = 0.70, ω = 0.69), which is comparable to prior research examining low base-rate and socially undesirable personality traits in non-clinical samples. With the exception of sadism, skewness and kurtosis values were within recommended thresholds for univariate normality [56].

The distribution of sadism was characterized by pronounced skewness and kurtosis, a pattern consistent with earlier findings on socially undesirable and low-frequency personality traits [29]. Such distributional characteristics are commonly observed in general population samples and reflect the rarity of elevated sadistic tendencies. To accommodate this non-normality, robust estimation methods and bootstrapped confidence intervals were employed. Moreover, given the large sample size, deviations from univariate normality were not expected to substantially bias parameter estimates [56]. The results based on bootstrapped confidence intervals were consistent with the reported estimates. In addition, a sensitivity analysis was also conducted by re-estimating the model without the sadism variable; the overall pattern of results remained substantively unchanged, suggesting that the findings were not driven by the distributional properties of sadism. Considering the satisfactory composite reliability values, average variance extracted values above 0.40 were regarded as acceptable in line with previous methodological recommendations [57].

Problematic generative artificial intelligence use (PGAIU) was positively correlated with intolerance of uncertainty (*r* = .35, *p* < .001), vertical individualism (*r* = .41, *p* < .001), narcissism (*r* = .29, *p* < .001), Machiavellianism (*r* = .19, *p* < .001), and sadism (*r* = .12, *p* < .01). Intolerance of uncertainty was positively associated with horizontal individualism (*r* = .08, *p* < .05), vertical individualism (*r* = .20, *p* < .001), narcissism (*r* = .16, *p* < .001), Machiavellianism (*r* = .17, *p* < .001), psychopathy (*r* = .20, *p* < .001), and sadism (*r* = .12, *p* < .01). Furthermore, vertical individualism was positively correlated with narcissism (*r* = .45, *p* < .001), Machiavellianism (*r* = .25, *p* < .001), psychopathy (*r* = .18, *p* < .001), and sadism (*r* = .16, *p* < .001).

Differences between men and women in the study variables were examined using independent-samples t-tests (coded as women = 1, men = 2), with the results not displayed in a separate table. The findings indicated that male participants scored higher than female participants on psychopathy (t[674] = − 3.95, *p* < .001) and vertical individualism (t[674] = − 3.16, *p* < .01). While higher mean scores for men were also observed for PGAIU, narcissism, Machiavellianism, and sadism, and higher scores for women on intolerance of uncertainty, these differences were not statistically significant. These findings should be interpreted with caution, as full measurement invariance across sex was not supported.

### Common method bias

Following the recommendations of Podsakoff et al. [58], the possibility of common method bias was examined using Harman’s single-factor approach. To this end, all measured variables were subjected to an exploratory factor analysis employing principal component extraction with varimax rotation, while restricting the solution to one factor. The findings indicated that this factor explained 18.76% of the overall variance, suggesting that common method bias is unlikely to fully account for the observed associations; however, this approach cannot rule out its presence. In addition to Harman’s single-factor test, we conducted an additional diagnostic check by examining variance inflation factors (VIFs). All VIF values were below conventional thresholds (VIFs < 3), suggesting that multicollinearity was not a concern.

### Model testing

The path model demonstrated an acceptable to good fit across the total sample and sex groups based on commonly recommended criteria. For the total sample, model fit indices were satisfactory (χ² = 45.45, df = 10, *p* < .001, RMSEA = 0.07, 90% CI [0.05, 0.09], SRMR = 0.02, CFI = 0.98, GFI = 0.99). Similarly, the model exhibited adequate fit among men (χ² = 36.18, df = 9, *p* < .001, RMSEA = 0.09, 90% CI [0.06, 0.13], SRMR = 0.03, CFI = 0.97, GFI = 0.98) and women (χ² = 12.78, df = 11, *p* < .001, RMSEA = 0.02, 90% CI [0.00, 0.06], SRMR = 0.02, CFI = 0.99, GFI = 0.99). Overall, these findings indicate that the proposed model fit the data well across the total sample and sex groups.

**Fig. 2:**
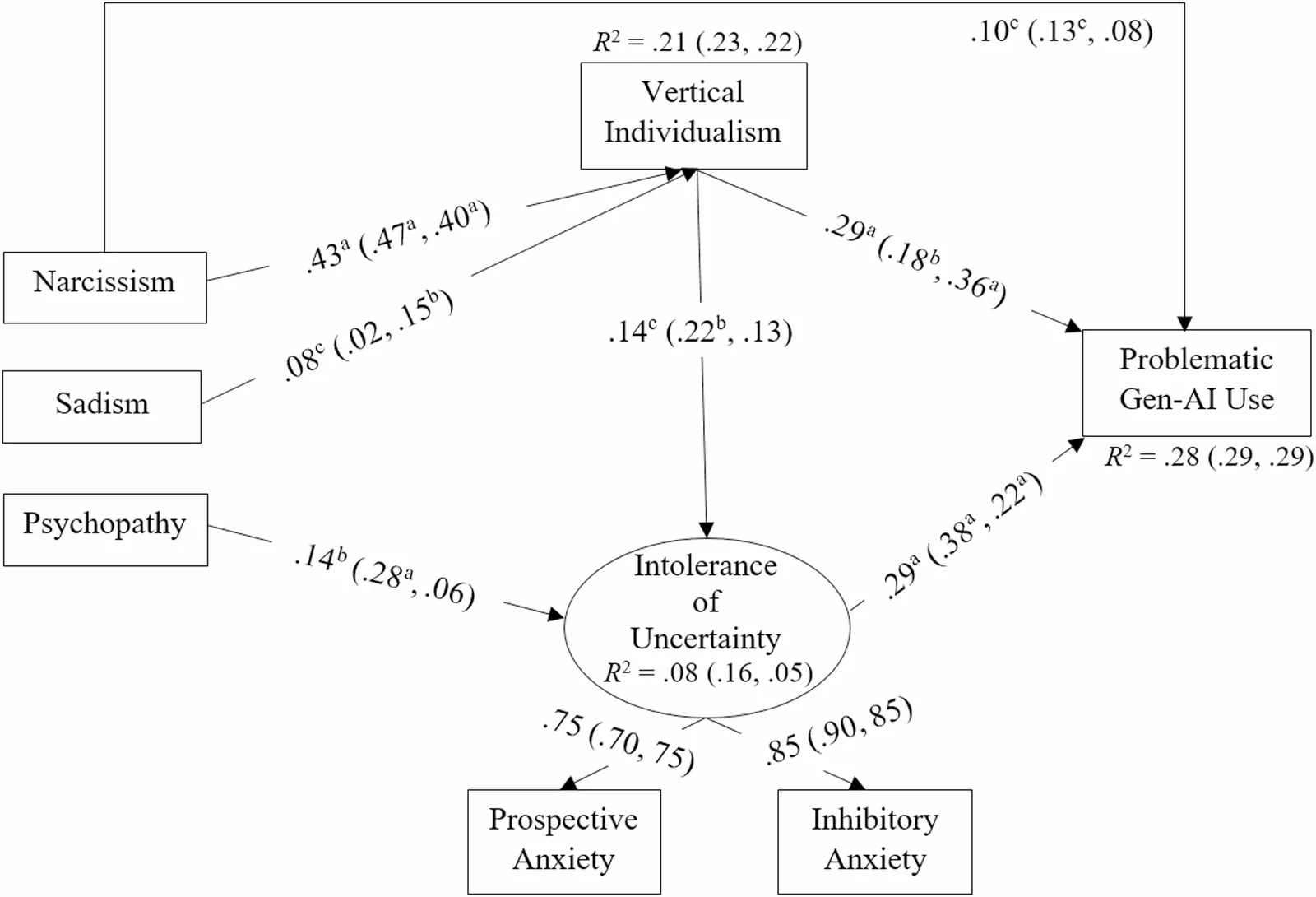
Final model of the significant path coefficients. Note. Latent constructs in the model are represented by circles, while observed indicators are illustrated as rectangles. Sex (coded as 1 = female, 2 = male; β= .05, p>.05; 95% CI [-.02, .11]), and age (β= -.10, *p*<.01; 95% CI [-.16, -.03]) were included in the model as control variables in total sample. Standardized path estimates are presented for the total sample outside the brackets; estimates for men and women are reported on the left and right sides of the brackets, respectively. For presentation purposes, several insignificant paths were omitted from the figure. ^c^*p* < .05, ^b^*p* < .01, ^a^*p* < .001

Table 3 presents the standardized total, direct, and indirect effects, and Fig. 2 illustrates the final path model. Narcissism exhibited a significant total effect on PGAIU in the total sample (*β* = 0.26, *p* < .001; 95% CI [0.17, 0.34]), as well as among men (*β* = 0.28, *p* < .001; 95% CI [0.16, 0.40]) and women (*β* = 0.23, *p* < .001; 95% CI [0.11, 0.36]). In the total sample and among men, narcissism showed both direct and indirect effects on PGAIU; however, the direct effect was non-significant among women (*β* = 0.08, *p* > .05; 95% CI [-0.06, 0.21]). Further analyses suggested that the association between narcissism and PGAIU was fully mediated via vertical individualism and vertical individualism–related intolerance of uncertainty in the total sample and among men. Vertical individualism was not associated with intolerance of uncertainty among women. The results remained substantively unchanged when controlling for country of residence using dummy variables, indicating that the observed associations involving individualism were not driven by between-country differences. These patterns should be interpreted with caution, as full measurement invariance across sex was not supported.

**Table 3:** Standardized estimates of total, direct, and indirect effects on problematic Gen-AI use for overall sample and men and women

	Effect (S.E.)		
	Total Sample (*N* = 677)	Men (*N* = 339)	Women (*N* = 337)
Narcissism➔ PGAIU (total effect)	0.26*** (0.04)	0.28*** (0.06)	0.23*** (0.06)
➔PGAIU (direct effect)	0.10* (0.04)	0.13* (0.06)	0.08 (0.07)
➔PGAIU (total indirect effect)	0.16*** (0.03)	0.15*** (0.04)	0.16*** (0.04)
➔VI➔PGAIU	0.12*** (0.01)	0.12** (0.01)	0.14*** (0.01)
➔VI➔IU➔PGAIU	0.02** (0.01)	0.04** (0.01)	0.01 (0.01)
Sadism➔PGAIU (total effect)	0.08* (0.04)	0.05 (0.06)	0.11 (0.06)
➔PGAIU (direct effect)	0.05 (0.03)	0.03 (0.05)	0.06 (0.05)
➔PGAIU (total indirect effect)	0.03 (0.02)	0.03 (0.02)	0.05 (0.03)
VI➔PGAIU (total effect)	0.33*** (0.05)	0.27*** (0.07)	0.39*** (0.07)
➔PGAIU (direct effect)	0.29*** (0.04)	0.18** (0.06)	0.36*** (0.06)
➔PGAIU (total indirect effect)	0.04* (0.02)	0.08** (0.03)	0.03 (0.02)

Only the significant effects are shown in the table; full report is available upon request

*PGAIU *Problematic Gen-AI Use, *VI *Vertical individualism, *IU*  Intolerance of uncertainty

*p < .05, **p < .01, ***p < .001

Sadism demonstrated a small but significant positive total effect on PGAIU (*β* = 0.08, *p* < .05; 95% CI [0.00, 0.17]). Nevertheless, neither its direct effect (*β* = 0.05, *p* > .05; 95% CI [-0.02, 0.12]) nor its indirect effect (*β* = 0.03, *p* > .05; 95% CI [-0.01, 0.07]) reached statistical significance, suggesting that its overall influence was not driven by specific pathways in the model. Additional robustness analyses indicated that the overall pattern of results remained substantively unchanged when controlling for country of residence; however, the total effect of sadism on PGAIU was attenuated and no longer statistically significant under this specification, suggesting that this association may be influenced by between-country differences. In contrast, psychopathy was found to be directly and negatively associated with PGAIU in the total sample, despite showing a non-significant bivariate correlation. This pattern suggests the presence of a suppression effect within the path model. Given the discrepancy between the non-significant bivariate correlation and the negative direct path in the multivariable model, the psychopathy finding was treated as a potential suppression effect. All VIF values were below conventional thresholds (VIFs < 3), suggesting that multicollinearity was not a concern. However, this result should still be interpreted with caution. Additional model re-specifications indicated that the psychopathy coefficient was sensitive to model composition, becoming weaker and non-significant when certain Dark Tetrad predictors were removed (particularly Machiavellianism).

Furthermore, psychopathy was directly positively related to intolerance of uncertainty among total sample (*β* = 0.14, *p* < .01; 95% CI [0.04, 0.25]) and men (*β* = 0.28, *p* < .001; 95% CI [0.12, 0.43]), but not women (*β* = 0.06, *p* > .05; 95% CI [-0.07, 0.21]). Finally, vertical individualism was both directly and indirectly related to PGAIU in the total sample and among men. However, its indirect association was not significant among women, suggesting potential sex-specific patterns in the underlying mechanisms. These patterns should be interpreted with caution, as full measurement invariance across sex was not supported.

As illustrated in Fig. 2, the proposed path model explained a considerable proportion of variance in PGAIU (*R*² = 0.28). Furthermore, the personality variables included in the model accounted for meaningful variance in vertical individualism (*R*² = 0.21) and intolerance of uncertainty (*R*² = 0.08). While adjusting for sex did not substantially change the observed associations, age was found to be modestly but significantly negatively related to PGAIU (*β* = −0.10, *p* < .01; 95% CI [-0.16, − 0.03]).

## Discussion

Generative artificial intelligence (GAI) has rapidly become a major topic of interest across individual and institutional settings [1]. The present study aimed to extend existing knowledge by examining the associations between problematic GAI use (PGAIU), personality traits, individualism, and intolerance of uncertainty (IU) in a Western community sample. Specifically, we assessed the direct and indirect associations of narcissism, Machiavellianism, psychopathy, and sadism with PGAIU via individualism and IU.

The findings indicated that vertical individualism and IU were consistently and positively related to higher levels of PGAIU. The association between narcissism and PGAIU appeared to be fully mediated by vertical individualism and IU among women, and partially mediated among men and in the total sample. Sadism demonstrated a significant total association with PGAIU, whereas Machiavellianism and horizontal individualism were not associated with PGAIU. Psychopathy showed a direct negative association with PGAIU in the model, despite a non-significant bivariate correlation. This pattern may reflect a suppression effect within the path model (i.e., another variable correlated with PGAIU that alters the association with psychopathy). Additional analyses indicated that this finding was partially dependent on model specification, suggesting that it may not represent a stable substantive association. However, we cannot rule out the possibility that this result reflects a statistical artifact [59]; therefore, the findings related to psychopathy should be interpreted with caution. Given that full measurement invariance across sex was not supported, sex-based comparisons should be interpreted cautiously, and the observed patterns should not be considered definitive evidence of group differences. Finally, given the relatively low mean levels of PGAIU and the positively skewed distribution, these associations may primarily reflect variability within generally low levels of problematic engagement rather than pronounced problematic use. In line with recent critiques, caution is warranted to avoid overpathologizing intensive or habitual GAI use in the absence of clear evidence of functional impairment or clinically significant harm [60].

As anticipated, IU was positively associated with higher levels of PGAIU across all groups. This finding aligns with prior research showing a positive link between IU and various forms of problematic digital use, such as smartphone addiction and problematic social media use [41, 42]. Theoretically, individuals with elevated IU may rely on increased technology use as a maladaptive coping strategy to reduce the negative affect associated with ambiguity [43]. Because GAI provides instantaneous responses and actionable solutions [2], it may function as an “uncertainty reduction” tool for individuals who are highly sensitive to uncertainty [44]. In line with the I-PACE framework, repeated attempts to manage distress through GAI may gradually foster cognitive and emotional reliance, which may be reflected in higher levels of PGAIU [12].

Notably, the association between IU and PGAIU appeared to be moderate among men and weaker among women. This pattern is broadly consistent with prior evidence indicating that men tend to report lower levels of IU than women [48], which may contribute to greater variability in IU-related outcomes among men in this sample. However, these patterns should be interpreted with caution, as measurement invariance across sex was not fully supported. The underlying mechanisms were not directly measured in this study and should be examined in future research using designs that can identify motivational mediators. Future research should further explore sex-specific processes that may help explain how IU relates to problematic GAI use.

Partially in line with expectations, vertical individualism, but not horizontal individualism, was positively associated with higher levels of PGAIU. This finding is consistent with previous research linking vertical orientations to internet addiction [32], although it differs from studies reporting similar associations for general individualism [31, 33]. In the context of GAI, it is plausible that vertical individualism—characterized by competitiveness and self-focused achievement [26]—is more strongly related to PGAIU than horizontal individualism. Features of GAI, such as rapid content generation, may be particularly appealing for individuals seeking competitive advantages [24].

Interestingly, the direct association between vertical individualism and PGAIU appeared to be moderate among women and weaker among men (partially mediated by higher IU among men), despite men reporting higher overall levels of vertical individualism. One possible explanation—though not directly tested in this study—is that individualistic orientations may operate differently across sex due to broader social contextual factors [61]. However, these patterns should be interpreted with caution, as measurement invariance across sex was not fully supported. Vertical individualism was not significantly associated with IU among women, suggesting that the pathway from individualism to PGAIU may not involve cognitive-affective amplification via IU in this group. Whether this reflects a more direct, goal-oriented engagement with GAI among women high in individualism remains speculative and warrants further investigation. The observed association between vertical individualism and IU among men may reflect differences in access to or use of supportive resources. For instance, individuals higher in individualism may place greater emphasis on self-reliance and thus be less inclined to seek support from others [14]. In contrast, some evidence suggests that women may maintain broader social networks that provide emotional buffering in the face of uncertainty [62].

As partially expected, narcissism showed a positive direct association with PGAIU in the total sample and among men. To our knowledge, this study is among the first to empirically examine this link in the context of PGAIU. This pattern is consistent with prior findings indicating that narcissistic tendencies are related to problematic engagement with various digital platforms and online tools [21]. Although narcissism can sometimes be associated with increased motivation, ambition, and performance-focused behavior [53], these same characteristics may also be linked to higher levels of GAI engagement. In this context, individuals high in narcissism may use GAI as a means of gaining status-related advantages, outperforming others, or showcasing competence in work or academic settings [46]. However, these patterns should be interpreted with caution, as measurement invariance across sex was not fully supported.

Narcissism was also indirectly associated with PGAIU via vertical individualism and its link to IU (i.e., serial mediation) in the total sample and among men. However, among women, the indirect association appeared to operate only through vertical individualism. The positive association between narcissism and vertical individualism across groups is consistent with prior research indicating that individuals high in narcissism tend to pursue agentic goals and social ranking to maintain a sense of superiority [27]. In particular, core features of narcissism—such as grandiosity and entitlement—align with the competitive and status-oriented aspects of vertical individualism [13]. However, these patterns should be interpreted with caution, as measurement invariance across sex was not fully supported.

The total association of sadism with PGAIU was significant. This is consistent with perspectives that characterize sadistic individuals as competitive actors who seek to assert dominance and importance [29]. Their vulnerability to higher levels of PGAIU may be linked to the efficiency of GAI in enabling users to influence others quickly [25]. Sadism was also positively associated with vertical individualism in the total sample and particularly among women. Vertical individualism may provide a socially acceptable pathway through which individuals high in sadism pursue goals related to power and hierarchy [63]. The competitive features of vertical individualism align with the tendency to outperform others, with status-oriented motives potentially serving as a channel for exerting interpersonal influence [29]. Finally, among the Dark Tetrad traits, psychopathy was positively associated with IU in the total sample and among men, but not among women. Importantly, additional analyses indicated that the positive association between psychopathy and IU remained consistent even when controlling for or removing other dark traits, suggesting that it reflects a unique relationship rather than shared variance. This finding is consistent with studies suggesting that psychopathic traits may be linked to higher intolerance of uncertainty [37], while contrasting with research associating psychopathy with low anxiety and fearlessness [64]. These patterns should be interpreted with caution, as measurement invariance across sex was not fully supported.

### Limitations and conclusion

Several limitations should be taken into account when interpreting the present findings. The study relied on volunteers who completed self-report questionnaires, which may not fully represent the wider population and may also be influenced by inaccurate or biased responding. To enhance confidence in future conclusions, researchers could incorporate additional forms of assessment, such as behavioral indicators, interview-based methods, or mixed-method approaches in larger and more heterogeneous samples. Furthermore, because the data were gathered at only one time point, the temporal ordering among the studied variables remains unclear. All variables were assessed using self-report measures at a single time point, which may increase the risk of common method bias; therefore, the findings should be interpreted with caution. Studies that track individuals across multiple waves or employ longitudinal designs will be needed to examine how these psychological characteristics and behaviors influence one another over time. The present sample consisted primarily of English-speaking adults from Western societies, limiting the generalizability of the findings to other cultural, linguistic, and demographic contexts. Replications in more diverse populations are therefore needed. Measurement invariance across sex was not fully supported, which limits the interpretability of cross-group comparisons; accordingly, sex-based differences should be interpreted with caution, and future studies should establish invariance before drawing firm conclusions.

A further limitation concerns the adaptation of the PGAIUS items from “conversational AI” to “GAI”. Although the wording changes were minimal and a CFA supported the factor structure in the present sample, no separate pretesting of the adapted items was conducted. Therefore, the results should be interpreted with caution, and future studies are encouraged to further validate the adapted scale across different samples and contexts. In addition, although the supplementary descriptive analyses provided greater transparency regarding the distribution of PGAIU scores, no additional ordinal or item-response-based robustness analyses were conducted. Future studies should examine whether the scale provides sufficient measurement precision across higher levels of the construct. Finally, the sadism variable exhibited substantial non-normality, and although bootstrapping was applied to mitigate its impact, this approach may not fully account for distributional violations; future studies should consider alternative estimation methods or modeling strategies for such variables.

Despite these limitations, the present study provides preliminary evidence, within this sample and with these measures, for direct and indirect associations involving PGAIU that have received limited attention in prior research. The findings suggest that dark personality traits may be associated with higher levels of PGAIU through motivational and cognitive-affective pathways, although replication in independent samples using longitudinal designs is needed before stronger conclusions can be drawn. Future work would benefit from incorporating more fine-grained assessments of these traits, such as vulnerable narcissism, subdimensions of narcissism, second-order psychopathy, and broader antisocial dispositions. Moreover, the observed associations involving vertical individualism and intolerance of uncertainty suggest that motivational orientations and cognitive-affective processes may be relevant to understanding GAI use patterns in this sample, and warrant further investigation in diverse populations. Together, these results highlight the need for continued research into how personality, motivational orientations, and sociocultural characteristics interact to shape patterns of GAI use across diverse populations, in order to better understand the rapidly evolving role of GAI in everyday life.

## Supplementary Information

Supplementary Material 1.

## Data Availability

The datasets generated and/or analyzed during the current study are available from the corresponding author upon reasonable request.

## Abbreviations

Gen-AI: Generative Artificial Intelligence
GAI: Generative Artificial Intelligence
PGAIU: Problematic Generative Artificial Intelligence Use
IU: Intolerance of Uncertainty
VI: Vertical Individualism
HI: Horizontal Individualism
CFI: Comparative Fit Index
GFI: Goodness-of-Fit Index
RMSEA: Root Mean Square Error of Approximation
SRMR: Standardized Root Mean Square Residual
