# Supplementary material for: Dark Tetrad traits and problematic generative artificial intelligence use: the serial mediating role of individualism and intolerance of uncertainty
Source: BMC Psychol. 2026 Jun 13;14:1220. doi: 10.1186/s40359-026-05012-4 (PMC13491676; doi:10.1186/s40359-026-05012-4)
Supplement: Supplementary file 1 [file 40359_2026_5012_MOESM1_ESM.docx]

**Supplementary Material S1. Distributional properties of PGAIU**
This section reports item-level response frequencies and the distribution of the total PGAIU score. The results show that responses are generally concentrated at the lower end of the scale; however, all response categories (1–5) are represented across items, with observable proportions of higher-category endorsements, indicating meaningful variability in the construct.

| **İtem Statistics** | | | | | | | |
| --- | --- | --- | --- | --- | --- | --- | --- |
|  | | pgaiu1 | pgaiu2 | pgaiu3 | pgaiu4 | pgaiu5 | pgaiu6 |
| N | Valid | 677 | 677 | 677 | 677 | 677 | 677 |
|  | Missing | 0 | 0 | 0 | 0 | 0 | 0 |
| Std. Deviation | | 1,051 | 1,206 | 1,079 | ,902 | ,929 | ,753 |
| Skewness | | ,515 | ,266 | 1,124 | 1,542 | 1,802 | 2,387 |
| Std. Error of Skewness | | ,094 | ,094 | ,094 | ,094 | ,094 | ,094 |
| Kurtosis | | -,346 | -,844 | ,423 | 1,825 | 2,638 | 5,532 |
| Std. Error of Kurtosis | | ,188 | ,188 | ,188 | ,188 | ,188 | ,188 |
| Minimum | | 1 | 1 | 1 | 1 | 1 | 1 |
| Maximum | | 5 | 5 | 5 | 5 | 5 | 5 |

| **pgaiu1** | | | | | |
| --- | --- | --- | --- | --- | --- |
|  | | Frequency | Percent | Valid Percent | Cumulative Percent |
| Valid | 1 | 177 | 26,1 | 26,1 | 26,1 |
|  | 2 | 231 | 34,1 | 34,1 | 60,3 |
|  | 3 | 183 | 27,0 | 27,0 | 87,3 |
|  | 4 | 66 | 9,7 | 9,7 | 97,0 |
|  | 5 | 20 | 3,0 | 3,0 | 100,0 |
|  | Total | 677 | 100,0 | 100,0 |  |

| **pgaiu2** | | | | | |
| --- | --- | --- | --- | --- | --- |
|  | | Frequency | Percent | Valid Percent | Cumulative Percent |
| Valid | 1 | 171 | 25,3 | 25,3 | 25,3 |
|  | 2 | 151 | 22,3 | 22,3 | 47,6 |
|  | 3 | 208 | 30,7 | 30,7 | 78,3 |
|  | 4 | 102 | 15,1 | 15,1 | 93,4 |
|  | 5 | 45 | 6,6 | 6,6 | 100,0 |
|  | Total | 677 | 100,0 | 100,0 |  |

| **pgaiu3** | | | | | |
| --- | --- | --- | --- | --- | --- |
|  | | Frequency | Percent | Valid Percent | Cumulative Percent |
| Valid | 1 | 348 | 51,4 | 51,4 | 51,4 |
|  | 2 | 157 | 23,2 | 23,2 | 74,6 |
|  | 3 | 110 | 16,2 | 16,2 | 90,8 |
|  | 4 | 43 | 6,4 | 6,4 | 97,2 |
|  | 5 | 19 | 2,8 | 2,8 | 100,0 |
|  | Total | 677 | 100,0 | 100,0 |  |

| **pgaiu4** | | | | | |
| --- | --- | --- | --- | --- | --- |
|  | | Frequency | Percent | Valid Percent | Cumulative Percent |
| Valid | 1 | 424 | 62,6 | 62,6 | 62,6 |
|  | 2 | 145 | 21,4 | 21,4 | 84,0 |
|  | 3 | 76 | 11,2 | 11,2 | 95,3 |
|  | 4 | 25 | 3,7 | 3,7 | 99,0 |
|  | 5 | 7 | 1,0 | 1,0 | 100,0 |
|  | Total | 677 | 100,0 | 100,0 |  |

| **pgaiu5** | | | | | |
| --- | --- | --- | --- | --- | --- |
|  | | Frequency | Percent | Valid Percent | Cumulative Percent |
| Valid | 1 | 457 | 67,5 | 67,5 | 67,5 |
|  | 2 | 122 | 18,0 | 18,0 | 85,5 |
|  | 3 | 58 | 8,6 | 8,6 | 94,1 |
|  | 4 | 30 | 4,4 | 4,4 | 98,5 |
|  | 5 | 10 | 1,5 | 1,5 | 100,0 |
|  | Total | 677 | 100,0 | 100,0 |  |

| **pgaiu6** | | | | | |
| --- | --- | --- | --- | --- | --- |
|  | | Frequency | Percent | Valid Percent | Cumulative Percent |
| Valid | 1 | 528 | 78,0 | 78,0 | 78,0 |
|  | 2 | 86 | 12,7 | 12,7 | 90,7 |
|  | 3 | 43 | 6,4 | 6,4 | 97,0 |
|  | 4 | 16 | 2,4 | 2,4 | 99,4 |
|  | 5 | 4 | ,6 | ,6 | 100,0 |
|  | Total | 677 | 100,0 | 100,0 |  |

| **Total score Statistics** | | |
| --- | --- | --- |
| PGAIU | | |
| N | Valid | 677 |
|  | Missing | 0 |
| Std. Deviation | | ,75572 |
| Skewness | | ,986 |
| Std. Error of Skewness | | ,094 |
| Kurtosis | | ,778 |
| Std. Error of Kurtosis | | ,188 |
| Minimum | | 1,00 |
| Maximum | | 5,00 |

| **PGAIU** | | | | | |
| --- | --- | --- | --- | --- | --- |
|  | | Frequency | Percent | Valid Percent | Cumulative Percent |
| Valid | 1,00 | 111 | 16,4 | 16,4 | 16,4 |
|  | 1,17 | 62 | 9,2 | 9,2 | 25,6 |
|  | 1,33 | 56 | 8,3 | 8,3 | 33,8 |
|  | 1,50 | 66 | 9,7 | 9,7 | 43,6 |
|  | 1,67 | 52 | 7,7 | 7,7 | 51,3 |
|  | 1,83 | 52 | 7,7 | 7,7 | 58,9 |
|  | 2,00 | 51 | 7,5 | 7,5 | 66,5 |
|  | 2,17 | 44 | 6,5 | 6,5 | 73,0 |
|  | 2,33 | 41 | 6,1 | 6,1 | 79,0 |
|  | 2,50 | 29 | 4,3 | 4,3 | 83,3 |
|  | 2,67 | 24 | 3,5 | 3,5 | 86,9 |
|  | 2,83 | 22 | 3,2 | 3,2 | 90,1 |
|  | 3,00 | 14 | 2,1 | 2,1 | 92,2 |
|  | 3,17 | 16 | 2,4 | 2,4 | 94,5 |
|  | 3,33 | 8 | 1,2 | 1,2 | 95,7 |
|  | 3,50 | 7 | 1,0 | 1,0 | 96,8 |
|  | 3,67 | 6 | ,9 | ,9 | 97,6 |
|  | 3,83 | 6 | ,9 | ,9 | 98,5 |
|  | 4,00 | 5 | ,7 | ,7 | 99,3 |
|  | 4,17 | 1 | ,1 | ,1 | 99,4 |
|  | 4,50 | 1 | ,1 | ,1 | 99,6 |
|  | 4,67 | 2 | ,3 | ,3 | 99,9 |
|  | 5,00 | 1 | ,1 | ,1 | 100,0 |
|  | Total | 677 | 100,0 | 100,0 |  |

**Supplementary Material S2. Sensitivity of psychopathy coefficient across model specifications**

| **Model specification** | **Psychopathy → PGAIU (β)** |
| --- | --- |
| Full model | −.12* |
| Without narcissism | −.12* |
| Without Machiavellianism | −.07 (ns) |
| Without sadism | −.06 (ns) |

**Supplementary Material S3. Standardized path coefficients with and without country controls**

| **(Total effect)** | **Original Model (β)** | **With Country Controls (β)** |
| --- | --- | --- |
| Narcissism → PGAIU | .26*** | .27*** |
| Machiavellianism → PGAIU | .08 | .07 |
| Psychopathy → PGAIU | -.09 | -.08 |
| Sadism → PGAIU | .08* | .07 (ns) |
| Vertical Individualism → PGAIU | .33*** | .33*** |
| IU → PGAIU | .29*** | .29*** |

**Supplementary Material S4**. *Omitted path coefficients for overall sample and men and* women*.*

|  | Effect (S.E.) | | |
| --- | --- | --- | --- |
|  | Total Sample  *(N=677)* | Men  *(N=339)* | Women *(N=337)* |
| Narcissism 🡺 VI (direct effect) | .43*** | .47*** | .40*** |
| 🡺HI (direct effect) | -.05 | -.01 | -.06 |
| 🡺 IU (total effect) | .11* | .17** | .05 |
| 🡺 IU (direct effect) | .05 | .07 | .00 |
| 🡺 IU (indirect effect) | .06* | .10** | .05 |
| Sadism 🡺 VI (direct effect) | .08* | .00 | .15** |
| 🡺HI (direct effect) | .08* | .05 | .11* |
| 🡺 IU (total effect) | .04 | .08 | .00 |
| 🡺 IU (direct effect) | .02 | .07 | -.03 |
| 🡺 IU (indirect effect) | .02* | .01 | .03* |
| Psychopathy 🡺 VI (direct effect) | .01 | -.08 | .05 |
| 🡺HI (direct effect) | .06 | .09 | .04 |
| 🡺 IU (total effect) | .14** | .27** | .07 |
| 🡺 IU (direct effect) | .14** | .28*** | .06 |
| 🡺 IU (indirect effect) | .00 | -.01 | .01 |
| Machiavellianism 🡺 VI (direct effect) | .03 | .07 | .01 |
| 🡺HI (direct effect) | -.07 | -.01 | -.13 |
| 🡺 IU (total effect) | .02 | -.10 | .11 |
| 🡺 IU (direct effect) | .02 | -.12 | .12 |
| 🡺 IU (indirect effect) | .00 | .02 | -.01 |
| VI 🡺 IU (direct effect) | .15* | .22** | .13 |
| HI 🡺 PGAIU (total effect) | -.05 | -.05 | -.03 |
| 🡺 PGAIU (direct effect) | -.07 | -.08 | -.04 |
| 🡺 PGAIU (indirect effect) | .02 | .03 | .01 |
| 🡺 IU (direct effect) | .07 | .08 | .06 |
| IU 🡺 PGAIU (direct effect) | .29*** | .38*** | .22*** |
| Age 🡺 PGAIU (direct effect) | -.10* | -.09* | -.11* |

*Note.* PGAIU = Problematic Gen-AI Use; VI = Vertical individualism; HI = Horizontal individualism; IU = Intolerance of uncertainty. **p* < .05, ***p* < .01, ****p* < .001.
